# Elicitation Induced α-Amyrin Synthesis in *Tylophora indica* In Vitro Cultures and Comparative Phytochemical Analyses of In Vivo and Micropropagated Plants

**DOI:** 10.3390/plants13010122

**Published:** 2023-12-31

**Authors:** Jyoti Mamgain, Abdul Mujib, Yashika Bansal, Basit Gulzar, Nadia Zafar, Rukaya Syeed, Ali Alsughayyir, Yaser Hassan Dewir

**Affiliations:** 1 Cellular Differentiation and Molecular Genetics Section, Department of Botany, Jamia Hamdard, New Delhi 110062, India; jyotimamgain93@gmail.com (J.M.); yashikab333@gmail.com (Y.B.); basit.gulzar786@gmail.com (B.G.); nadiawar20@gmail.com (N.Z.); rukayasyeed@gmail.com (R.S.); 2 Department of Plant and Soil Sciences, Mississippi State University, 75 B.S. Hood Rd, Starkville, MS 39762, USA; aa2942@msstate.edu; 3 Plant Production Department, College of Food and Agriculture Sciences, King Saud University, Riyadh 11451, Saudi Arabia; ydewir@ksu.edu.sa

**Keywords:** α-amyrin, biochemical analyses, elicitors, GC–MS analysis, HPTLC, in vitro culture, *Tylophora indica*

## Abstract

*Tylophora indica* (Burm. f.) Merrill is an endangered medicinal plant that possesses various active agents, such as tylophorinine, kaempferol, quercetin, α-amyrin and beta-sitosterol, with multiple medicinal benefits. α-amyrin, a triterpenoid, is widely known for its antimicrobial, anti-inflammatory, gastroprotective and hepatoprotective properties. In this study, we investigated the metabolite profiling of tissues and the effects of cadmium chloride and chitosan on in vitro accumulation of alkaloids in *T. indica.* First, the callus was induced from the leaf in 2,4-D-, NAA- and/or BAP-fortified MS medium. Subsequent shoot formation through organogenesis and in vitro roots was later induced. Gas chromatography–mass spectrometry (GC–MS)-based phytochemical profiling of methanolic extracts of in vivo and in vitro regenerated plants was conducted, revealing the presence of the important phytocompounds α-amyrin, lupeol, beta-sitosterol, septicine, tocopherol and several others. Different in vitro grown tissues, like callus, leaf and root, were elicited with cadmium chloride (0.1–0.4 mg L^−1^) and chitosan (1–50 mg L^−1^) to evaluate the effect of elicitation on α-amyrin accumulation, measured with high-performance thin layer chromatography (HPTLC). CdCl_2_ and chitosan showed improved sugar (17.24 and 15.04 mg g^−1^ FW, respectively), protein (10.76 and 9.99 mg g^−1^ FW, respectively) and proline (7.46 and 7.12 mg g^−1^ FW), especially at T3 (0.3 and 25 mg L^−1^), in the leaf as compared to those of the control and other tissues. The antioxidant enzyme activities were also evaluated under an elicitated stress situation, wherein catalase (CAT), superoxide dismutase (SOD) and ascorbate peroxidase (APX) displayed the highest activities in the leaf at T4 of both of the two elicitors. The α-amyrin yield was quantified with HPTLC in all tested tissues (leaf, callus and root) and had an Rf = 0.62 at 510 nm wavelength. Among all the concentrations tested, the T3 treatment (0.3 mg L^−1^ of cadmium chloride and 25 mg L^−1^ of chitosan) had the best influence on accumulation, irrespective of the tissues, with the maximum being in the leaf (2.72 and 2.64 μg g^−1^ DW, respectively), followed by the callus and root. Therefore, these results suggest future opportunities of elicitors in scaling up the production of important secondary metabolites to meet the requirements of the pharmaceutical industry.

## 1. Introduction

Plants possess various biosynthetic pathways that synthesize a wide range of bioactive compounds from different precursors and primary metabolites [1]. The plants produce secondary metabolites in trace concentrations; the synthesis, however, depends upon different factors, such as physiological conditions, growth stages and environmental situations [2]. Different classes of secondary metabolites (SMs), such as phenolic, flavonoids, alkaloids, triterpenoids, saponins and steroids, are of great human interest as these serve as potent therapeutic drugs in the pharmaceutical industry [3]. These plant secondary metabolites are now being extensively utilized in herbal, cosmetic and nutraceutical formulations [4]. However, the over-exploitation of plants has led to a drastic decline of biodiversity [5]. In vitro techniques propagate plants at a much faster rate with no risk of pathogens, and this method can be used as a reliable tool for the high yield of SMs [6,7].

Phytochemical profiling with gas chromatography–mass spectrometry (GC–MS) has recently emerged as one of the most valuable techniques to detect and identify bioactive compounds, like alkaloids, flavonoids, tannins, phenols, steroids, terpenoids and essential oils [8,9]. This technique requires a low extract level to analyze metabolites with precision and reproducibility [10]. There are reports of GC–MS-based profiling of in vitro regenerated plants, like *Tecoma stans* [11] and *Curcuma zedoaria* [12]. The identification and characterization of phytocompounds with GC–MS may further auger the improvement of yield. To improve biomass production and bioactive compounds, the elicitation of tissues seems to be an important prominent approach. Its supremacy over other methods lies in the fact that the technique allows for the fast synthesis of phytochemicals with no seasonal dependence on yield [13]. Elicitation is an approach that enhances the production of SMs in culture; it also induces stress and promotes various biosynthetic activities that can later be utilized to understand the stress-responsive influence on the triggering of compound synthesis [14]. Elicitors are biotic or abiotic substances that increase the production of specific compounds when used at trace levels [15]. Abiotic elicitors are physical and chemical agents, like UV, high and low temperature, osmotic factors (sorbitol, mannitol, polyvinyl pyrrolidone, etc.), heavy metal salts (AgNO_3_, CdCl_2_, CuCl_2_, CuSO_4_, VOSO_4_, NiSO_4_, etc.) and intracellular signaling molecules, like salicylic acid, methyl jasmonate and systemin [16]. Biotic elicitors, on the other hand, are of biological origin from plants or microorganisms, such as yeast extract, pectin, chitin, chitosan and glucans [17]. The elicitation through biotic and abiotic compounds has frequently been applied to enhance SMs in diverse plant species [18,19,20].

*Tylophora indica* (Burm. f.) Merrill belongs to Asclepidaceae and is an important perennial woody plant with importance in the Ayurveda and Siddha systems of medicines [21]. The plant is known for its anti-cancerous, anti-inflammatory, antioxidant, anti-asthmatic, hypotensive and anti-convulsant activities. The plant parts of *T. indica* contain various secondary metabolites, such as alkaloids, flavonoids, terpenoids, phenols, saponins and sterols [22]. Among all of these classes, terpenoids have recently gained popularity as these impact plant reproduction, thermal tolerance and in-plant defense systems [23]. α-amyrin is one such important triterpenoid that possesses various pharmacological potentials, including anti-tumor, anti-inflammatory and hepatoprotective properties, and is present in several plant species [24].

The present study was undertaken to obtain a metabolite profiling with GC–MS and the detection of phytocompounds present in tissue culture-derived tissues. Two elicitors, i.e., cadmium chloride (abiotic) and chitosan (biotic), were applied in vitro to improve α-amyrin, and the yield was quantified with HPTLC. Different biochemical attributes were also checked in response to the elicitation treatments. Studies targeting α-amyrin using elicitation under in vitro conditions are, unfortunately, lacking; thus, this study is the first report on the enhanced production of α-amyrin in *T. indica*.

## 2. Results

### 2.1. Callus Induction and Organogenesis

**Figure 1:**
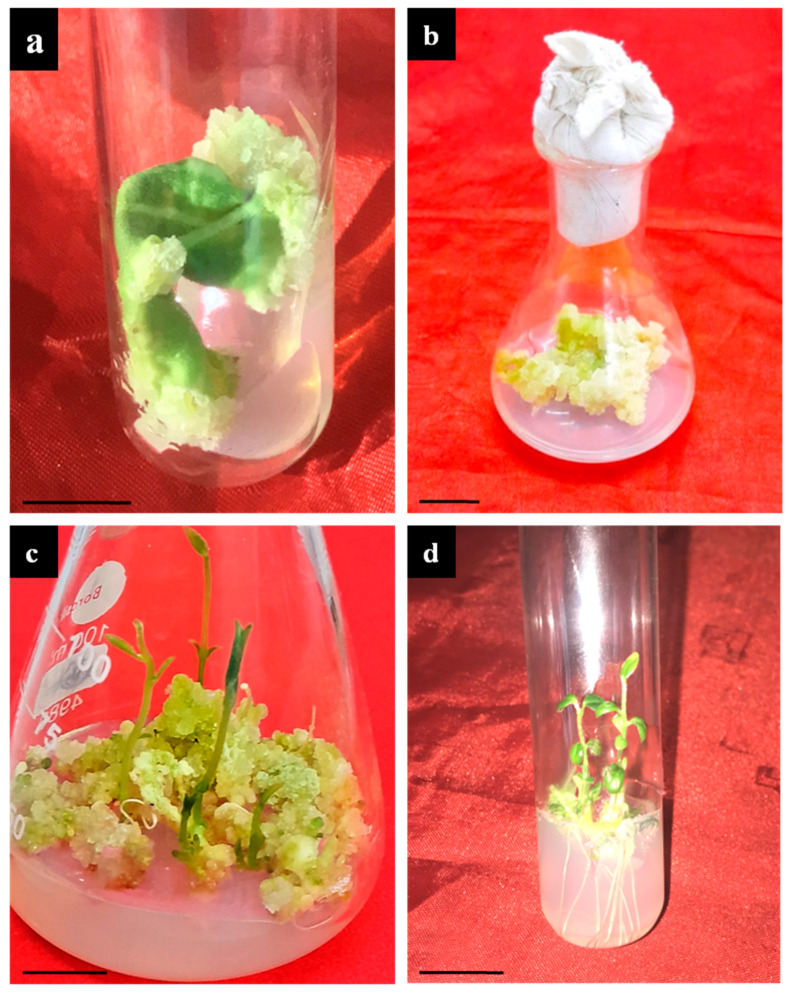
Callus-mediated indirect organogenesis from leaf-derived callus. (**a**) Callus induction from leaf explant, (**b**) callus proliferation (after 4 weeks of culture), (**c**) multiple shoot buds; regeneration medium contained 2.0 mg L^−1^ BAP + 0.5 mg L^−1^ NAA, (**d**) rooting of shootlets in 1.0 mg L^−1^ IBA (Bar (**a**): 1.5 cm, (**b**): 1.0 cm, (**c**): 2.0 cm, (**d**): 1.5 cm).

Leaves of *T. indica* were inoculated onto MS medium fortified with varied concentrations of 2,4-D, NAA and BAP to induce the callus (Table 1). Among all the tested treatments, 2,4-D alone at 1.5 mg L^−1^ proved to be the best at inducing the callus with a callusing frequency of 82.25%, whereas the callusing efficiency (20.11%) was the least on BAP-added (2 mg L^−1^) and NAA-added (0.5 mg L^−1^) medium (Figure 1a). With a further increase in BAP, no significant influence on callusing was observed. With time (4–5 weeks of inoculation), the callus proliferated more, and shoot regeneration from the callus was noticed (Figure 1b). The maximum shoots from the leaf–calli were obtained in the 2.0 mg L^−1^ BAP + 0.5 mg L^−1^ 2,4-D-added medium at a frequency of 68.15% with an average of 4.19 ± 0.15 shoot/callus mass (Figure 1c). At a higher level of BAP, no significant improvement in shoot formation was observed. The regenerated shoots were later transferred to medium for root induction.

### 2.2. Rooting and Acclimatization

The in vitro regenerated shoots were cultured onto root-induction media, supplemented with IBA and NAA (Table 2). The highest root induction (83.14%) was observed on 1.0 mg L^−1^ IBA-supplemented medium with 8.12 mean number of roots per shoot (Figure 1d). In contrast, NAA at 0.25 mg L^−1^ was least responsive in generating roots (31.52%). All other tested treatments showed moderate rooting on in vitro-derived shoots. These rooted shoots were successfully transplanted in outdoor pots and kept in a greenhouse with a survival rate of 75–80%.

### 2.3. GC–MS Analysis

**Figure 2:**
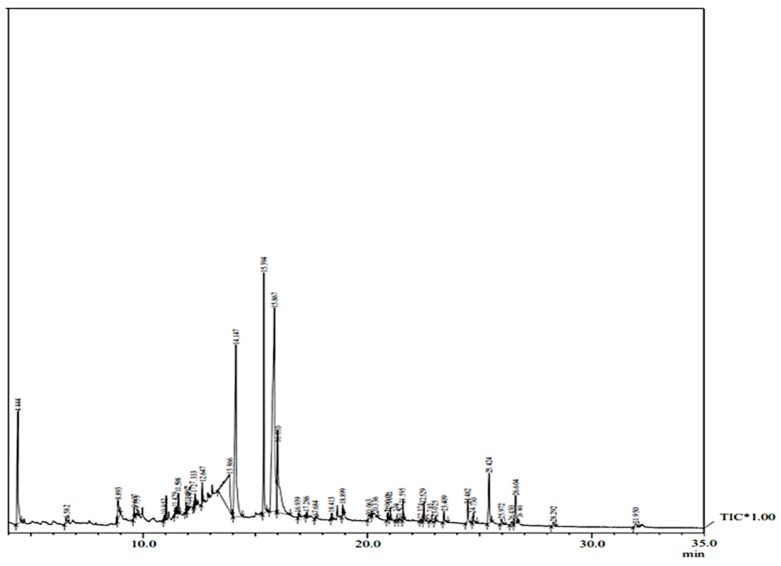
GC–MS chromatogram of methanolic leaf extract of in vivo plants of *T. indica*.

**Figure 3:**
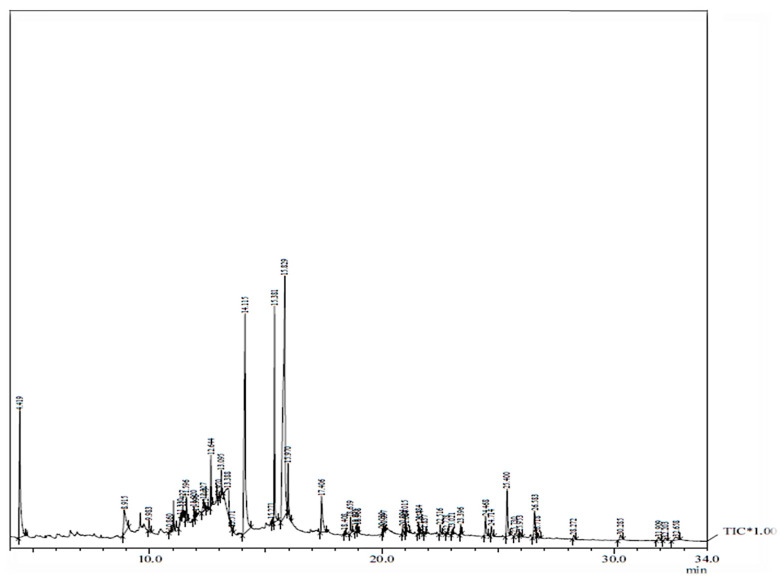
GC–MS chromatogram of methanolic leaf extract of in vitro-developed plants of *T. indica*.

The detection of the various phytocompounds present in the methanolic extracts of the in vitro-raised plants was performed with GC–MS and was compared with in vivo-grown *T. indica* plants. The GC–MS analysis involves the active principles with respective retention time (RT), peak area, area % and the spectra of unknown bioactive compounds and was compared with known compounds retrieved from the NIST (National Institute Standard and Technology) library (Figure 2 and Figure 3). The methanolic leaf extracts of both sources produced chromatograms showing the presence of over 40 phytocompounds that are listed in Table 3 and Table 4. Several phytocompounds were detected in trace amounts in both in vivo- and in vitro-grown plants, belonging to diverse classes, such as terpenoids, phenolics, alkaloids, saponins, tannins, etc. Some of the important bioactives identified in both the extracts are septicine, gamma-tocopherol, vitamin E, gamma-sitosterol, α-amyrin, lupeol, inositol, neophytadiene, phytol, stigmasta-5,22-dien-3-ol, etc.

### 2.4. Effect of Elicitor Treatments on Sugar, Proline and Protein Contents in In Vitro Cultures

**Figure 4:**
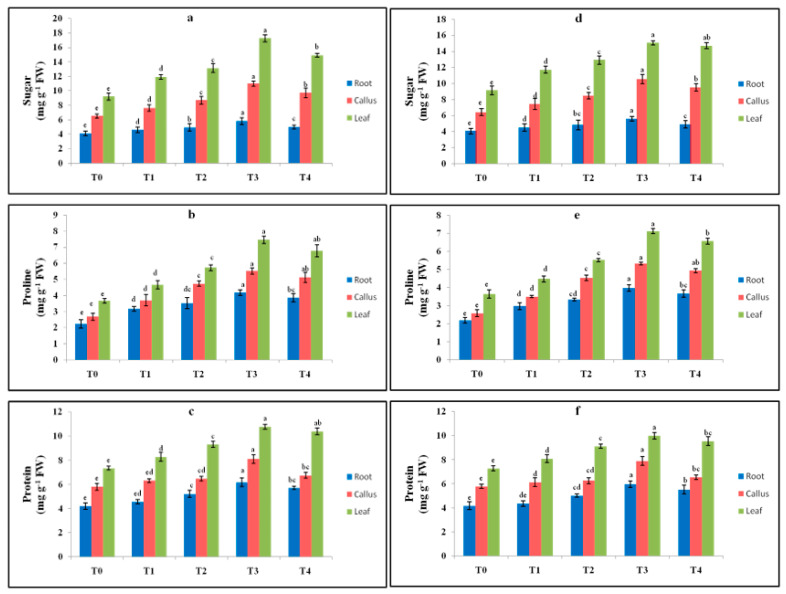
Sugar, protein and proline contents in mg g^−1^ FW in the root, callus and leaf parts of *T. indica* treated with different CdCl_2_ treatments (**a**–**c**) (T0: Control; T1: 0.1; T2: 0.2; T3: 0.3; T4: 0.4 mg L^−1^) and different chitosan treatments (**d**–**f**) (T0: Control; T1: 1; T2: 5; T3: 25; T4: 50 mg L^−1^). Values are expressed as means ± standard errors of three replicates. Means followed by same letters are significantly different at *p* ≤ 0.05 according to Duncan’s multiple range test (DMRT). T0: Control; T1: 0.1; T2: 0.2; T3: 0.3; T4: 0.4 mg L^−1^.

Cadmium chloride and chitosan were the elicitors applied to the media to induce stress. The in vitro-regenerated callus, leaves and roots were cultured on MS with varying concentrations of elicitors. The different tissues were exposed for two weeks, and the effects of the elicitation on biochemical parameters, like antioxidant enzymes, sugar, protein and proline, were investigated. With the increasing concentration of elicitor, the amount of sugar was also increased, the highest being in T3 (cadmium chloride and chitosan). The maximum sugar accumulation was 17.24 mg g^−1^ FW in the T3 treatment with CdCl_2_ in the leaf. In the callus and roots, the sugar content was 10.98 and 5.79 mg g^−1^ FW, respectively (Figure 4a). A similar trend with less accumulation was noticed in the chitosan-added medium, being the highest in the leaf, callus and root, i.e., 15.04, 10.53 and 5.59 mg g^−1^ FW, respectively, in the T3 treatment (Figure 4d).

The proline content was more on the 0.3 mg L^−1^ cadmium chloride-amended and 25 mg L^−1^ chitosan-amended conditions, which decreased at higher doses. In the CdCl_2_ treatment, the proline enhancement was noted to be higher in the tissues (leaf, callus and root), i.e., 7.46, 5.52 and 4.18 mg g^−1^ FW, respectively (Figure 4b). In the chitosan-added media, the maximum proline accumulation was 7.12 mg g^−1^ FW in the leaf (Figure 4e). In the callus and roots, the proline content observed was 5.32 and 3.98 mg g^−1^ FW, respectively.

The soluble protein was high on an elevated dosage of elicitors. The highest amount of protein was accumulated in T3 (cadmium chloride and chitosan), and the protein level declined at higher doses. In the leaf, the maximum protein accumulation, i.e., 10.76 mg g^−1^ FW, was observed in T3; it was 7.34 in the control, T0 (Figure 4c). Chitosan, on the other hand, had less influence with the highest being in the leaf, i.e., 9.99 mg g^−1^ FW, followed by the callus and root (Figure 4f).

### 2.5. Effect of Elicitor Treatments on Antioxidant Enzyme Activity

**Figure 5:**
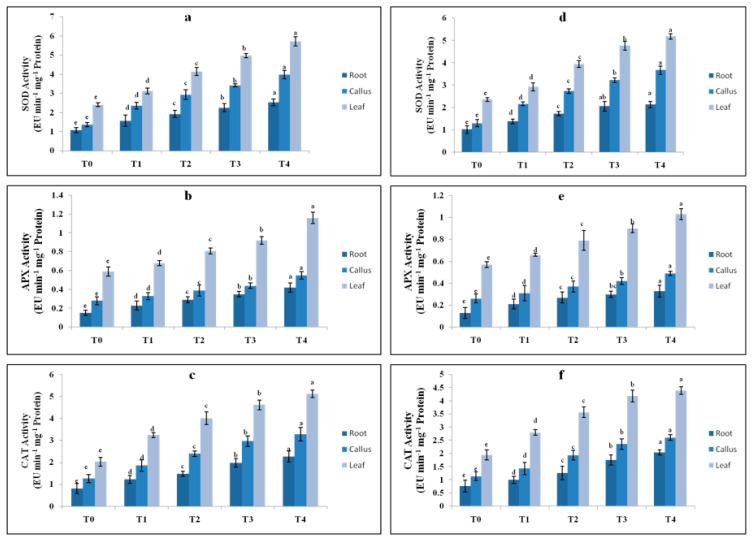
SOD, APX and CAT activities in EU mg^−1^ protein min^−1^ in the root, callus and leaf parts of *T. indica* treated with different CdCl_2_ treatments (**a**–**c**) (T0: Control; T1: 0.1; T2: 0.2; T3: 0.3; T4: 0.4 mg L^−1^) and different chitosan treatments (**d**–**f**) (T0: Control; T1: 1; T2: 5; T3: 25; T4: 50 mg L^−1^). Values are expressed as means ± standard errors of three replicates. Means followed by same letters are significantly different at *p* ≤ 0.05 according to Duncan’s multiple range test (DMRT).

In many earlier studies, elicitation-induced stress in culture was measured using stress markers like antioxidant enzyme activity. The SOD increased considerably as the elicitor level rose from T0 to T4. The maximum SOD (5.72 EU mg^−1^ protein min^−1^) was observed in the leaf, followed by the callus (3.98 EU mg^−1^ protein min^−1^) and root (2.53 EU mg^−1^ protein min^−1^), as compared to the control (Figure 5a). Chitosan was less responsive in increasing SOD, i.e., 5.18, 3.67 and 2.13 EU mg^−1^ protein min^−1^ in the leaf, callus and root, respectively (Figure 5d). The APX enzyme activity was highest at T4 (0.4 mg L^−1^) of CdCl_2_, i.e., 1.16 EU mg^−1^ protein min^−1^ in the leaf, 0.55 mg^−1^ protein min^−1^ in the callus and 0.42 mg^−1^ protein min^−1^ in the root, and was lowest at T0, i.e., 0.15 EU mg^−1^ protein min^−1^(Figure 5b). Similarly, chitosan at T4 displayed the maximum APX activity, i.e., 1.03 EU mg^−1^ protein min^−1^ in the leaf (Figure 5e). CAT activity was highest in the leaf at T4 of CdCl_2_ with 5.12 EU mg^−1^ protein min^−1^ protein and was lowest in the root (Figure 5c). On the other hand, chitosan had little influence in causing stress, as low CAT activity, i.e., 4.39 EU mg^−1^ protein min^−1^ activity, was noted at T4 (50 mg L^−1^) (Figure 5f).

### 2.6. Quantification of α-Amyrin in Different Tissues of T. indica with HPTLC

**Figure 6:**
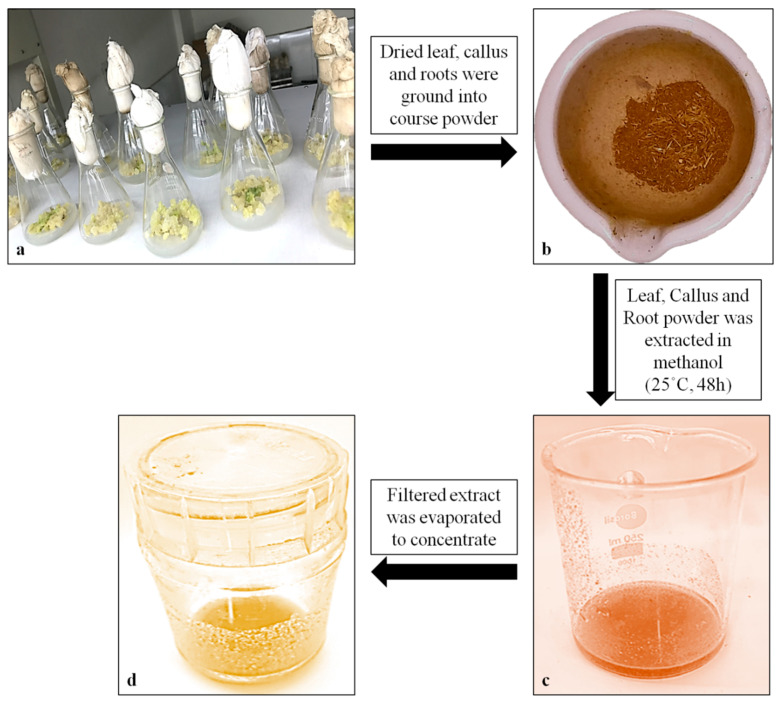
The steps followed for the preparation of the solvent extract in the *Tylophora* plant. (**a**) Small pieces of dried leaf, callus and roots; (**b**) coarse powder of dried tissues; (**c**) sample extract in methanol (at 25 °C for 48 h); (**d**) concentrate of methanolic sample extract.

**Figure 7:**
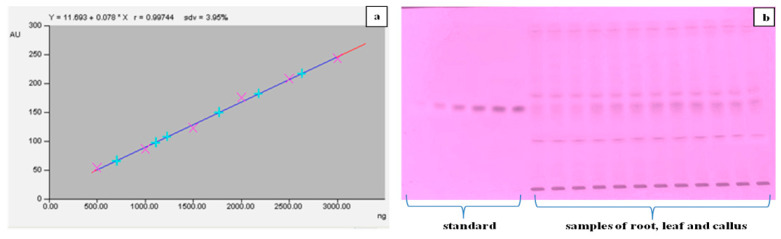
(**a**) The six-point calibration curve of α-amyrin with linear regression correlation coefficient r = 0.997 and regression equation y = 0.078 × x + 11.693, where y is the spot area and x is the concentration in ng/spot. (**b**) HPTLC separation of standard (α-amyrin) and leaf, callus and root samples of regenerated *T. indica*.

**Figure 8:**
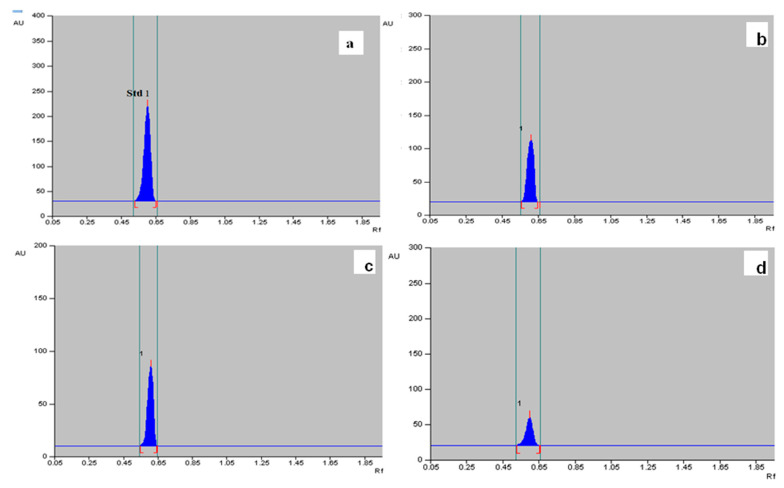
(**a**) HPTLC densitograms displaying single, sharp and flat peaks of the α-amyrin standard at Rf = 0.62, measured at wavelength = 580 nm; (**b**–**d**) show HPTLC densitograms of α-amyrin in leaf, callus and root extracts of field-grown plants displaying similar peaks at Rf = 0.62.

The methanolic extract of dried callus, leaves and roots of *T. indica* was prepared (Figure 6), and the α-amyrin yield was quantified using HPTLC. The six-point linear calibration of α-amyrin revealed good linearity with a regression correlation coefficient r = 0.998 and regression equation y = 11.693 + 0.078x, where y is the spot area and x is the concentration in μg/spot. The mobile phase used was toluene:ethyl acetate in the ratio of 9.5:0.5 with a saturation time of 1 h, which displayed a single sharp, flat and compact peak at Rf = 0.62, detected at wavelength 510 nm. The densitogram of the calibration curve and standard is shown in Figure 7 and Figure 8a. A higher amount of α-amyrin was noted in the cultures elicited with cadmium chloride and chitosan compared to the control (Figure 8b–d). Both cadmium chloride and chitosan showed the maximum accumulation of α-amyrin at T3, whereas T0 (control) had the least level (Table 5).

**Figure 9:**
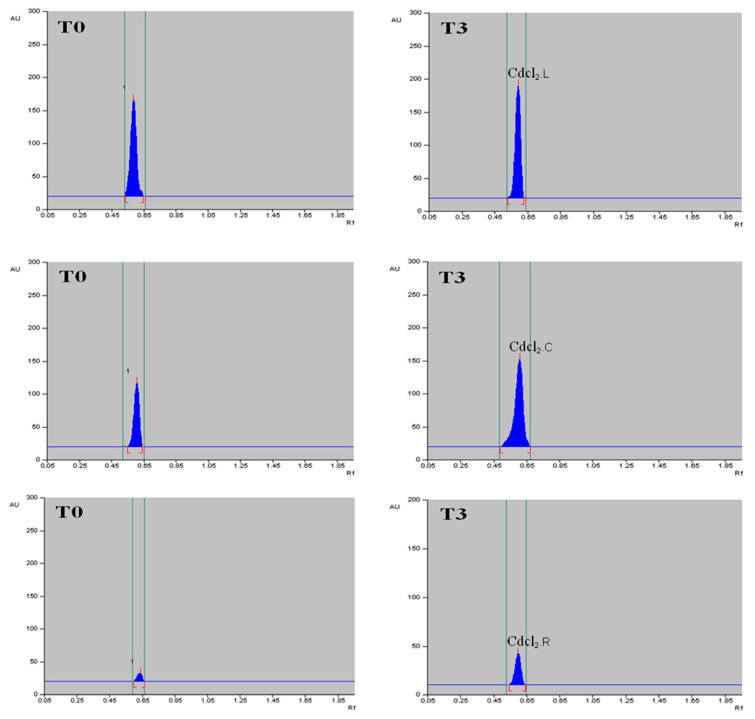
HPTLC densitograms of α-amyrin content of leaf (**top**), callus (**middle**) and root (**bottom**) tissues on control and on elicitation of T3 treatment of CdCl_2_ in *T. indica*.

**Figure 10:**
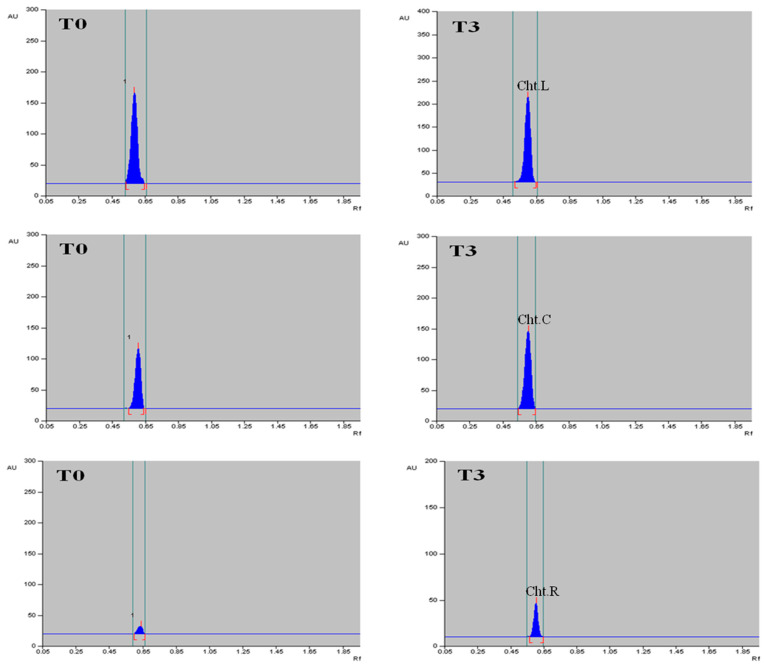
HPTLC densitograms of α-amyrin content of leaf (**top**), callus (**middle**) and root (**bottom**) tissues on control and on elicitation of T3 treatment of chitosan in *T. indica*.

The maximum content of α-amyrin (2.72 μg g^−1^ DW) was noted in the leaf with CdCl_2_ (Figure 9); α-amyrin was also found in the callus and roots, i.e., 1.51 and 0.68 μg g^−1^ DW, respectively. A similar trend was noticed with chitosan, wherein the leaf had maximum α-amyrin (2.64 μg g^−1^ DW) at T3 as compared to the control (1.67 μg g^−1^ DW), followed by the callus (1.45 μg g^−1^ DW) and root (0.61 μg g^−1^ DW) (Table 6, Figure 10). A gradual decline in α-amyrin accumulation was noted beyond the T3 treatment. Thus, the content of α-amyrin in the different tissues of *T. indica* under both elicitors is leaf > callus > root.

## 3. Discussion

*T. indica* is an important medicinal plant, extensively studied for various therapeutic activities, and is used in traditional medicine systems [25]. It is widely known because of the presence of a wide range of phytoconstituents, such as tylophorine, tylophorinine, kaempferol, quercetin, lupeol, α-amyrin and beta-amyrin [22]. The present study investigated the role of cadmium chloride and chitosan on the synthesis of α-amyrin, a medicinally important triterpenoid in *T. indica*. The experiment started with the establishment of the callus and subsequent organogenesis using leaf explants. Considering the positive influence of auxin alone or in combinations with cytokinins [26,27], callus formation was best noted on 1.5 mg L^−1^ 2,4-D-fortified MS medium. Similarly, a BAP (2.0 mg L^−1^) and NAA (0.5 mg L^−1^) combination was observed to be very efficient in shoot bud formation and proliferation. The promotive effect of NAA + BAP in the formation of shoots was reported in several plant species, like in *Artemisia annua* [28] and *Asparagus cochinchinensis* [29]. The rooting of regenerated shoots was obtained on IBA- or NAA-augmented MS medium in *Tylophora*; the same was described in other different plant species [30,31].

The in vitro culture conditions, temperature, photoperiod, PGR type and concentration and passaging duration induce stress in culture, which affect the biochemical and metabolite profiles of tissues [11]. Phytochemical characterization is an immensely valuable step in detecting and quantifying novel bioactive compounds from in vitro-derived tissues (callus, somatic embryo and other plant parts). The application of GC–MS-based chemical profiling for the recognition and documentation of a diverse array of phytochemicals has been exemplified in many scientific reports [32,33,34]. In the present study, the GC–MS-based comparative analysis of the metabolites of tissue culture and field-grown (mother) plants was conducted in order to obtain the phytochemical profiles of two alternative sources of *T. indica* plants. The investigation revealed the presence of over 40 phytocompounds in both in vivo- and in vitro-derived plant extracts. Among the diverse phytocompounds identified, α-amyrin and lupeol are the major triterpenoids, detected in both samples. Both possess diverse pharmacological activities, i.e., antibacterial, antifungal, anti-inflammatory, antioxidant and anti-cancerous properties [35,36]. Several other phytocompounds, like vitamin E, tocopherol, septicine, phytol and sitosterol, were also present in both extracts.

In vitro elicitation was conducted using one abiotic and one biotic elicitor (cadmium chloride and chitosan, respectively). Following elicitation, the biochemical analyses were conducted to examine the impact of the elicitors on the non-enzymatic and enzymatic attributes. With increasing cadmium chloride and chitosan dosages, the level of soluble carbohydrates increases in *T. indica*. The maximum increase in soluble sugar was noted at T3 of the cadmium chloride and chitosan treatments. Sugar accumulation in response to stress is a good indicator of a plant’s defensive mechanism as it leads to increased cellular osmolality [37]. The positive impact of elicitors on sugar accumulation was reported in several studied plants [38,39]. In response to an elicitor, soluble protein increased at various stress levels. One of the common adaptive mechanisms of osmotic adjustments and protection of cells from oxidative damage is to produce and accumulate more protein when the plants are under pressure [40]. Proline is a widely distributed osmolyte in plant cells [41]. Proline plays a crucial role in adjusting the osmotic balance of cells and, thereby, preserving the cell machinery under stress [42]. During our investigation, we observed that, with an incremental rise in cadmium chloride and chitosan in the medium, there was an enhanced proline accumulation in the tissues. A similar trend in proline content enhancement in stress environments was noted earlier in several studies [43,44]. Abiotic and biotic stress stimulated reactive oxygen species (ROS) production and increased antioxidant activity, such as CAT, SOD and APX, in response to oxidative damage [4]. Upon the excessive production of ROS, SOD activity is enhanced, which releases hydrogen peroxide (H_2_O_2_) as a by-product. This hydrogen peroxide is later scavenged by CAT and APX activities [45]. Thus, in the current study, a noticeable increase in enzyme activity was noted on amendment of cadmium chloride and chitosan dosages, and the levels of SOD, CAT and APX were found to be higher compared to those of the non-treated cultures. These observations suggest that elicitation could be a potent source of antioxidant modulation in plants. Therefore, the SOD–CAT–APX enzymatic machinery plays a defensive role against stress-induced oxidative damage, which is in accordance with the previously conducted investigations in various plant species [46,47].

Finally, the quantification of α-amyrin was performed with high-performance thin layer chromatography (HPTLC), regarded as one of the most sophisticated instrumental techniques for the qualitative and quantitative analysis of plant-based chemicals and drugs. Cadmium chloride and chitosan acted as exogenous elicitors; these compounds trigger a series of signal transduction pathways by upregulating stress-induced genes/proteins that, in turn, enhance the synthesis of secondary metabolites through transcriptional reprogramming and ‘elicitor–receptor complex’ formation [48]. In this experimental set-up, the α-amyrin increase was up to 2–3 folds compared to the control (non-elicited) in both elicitors. It was noted that the higher elicitor concentrations delimit the production of secondary metabolites, as observed in other HPTLC-targeted analyses in different plants [39,49]. Thus, we can conclude that both cadmium chloride and chitosan are promising elicitors in enhancing the yield of therapeutically active compounds present in plants. Till now, very little information of elicitation has been available in *T. indica* [50,51]. This is the first report of α-amyrin enhancement under abiotic and biotic stress in *T. indica*, and this enrichment possibility may be extended to other α-amyrin-producing plants for pharmaceutical application.

## 4. Materials and Methods

### 4.1. Explant Preparation and Culture Conditions

Young and healthy leaves of four-year-old *T. indica* were procured from the herbal garden, Jamia Hamdard, New Delhi and were used as the experimental material. The surface sterilization of the explants was conducted according to the earlier described protocol [7]. The disinfected leaves were then inoculated on autoclaved MS [52] medium comprising 3% (*w*/*v*) sucrose and 0.8% (*w*/*v*) agar augmented with different concentrations and combinations of PGRs (plant growth regulators), and the pH was adjusted to 5.7. The culture was maintained at a temperature of 24 ± 2 °C, a 16 h light photoperiod at an intensity of 40 µmol m^−2^ s^−1^ and 55–60% relative humidity.

### 4.2. Callus Induction and Indirect Organogenesis

Leaf explants were cultured onto callus induction medium enriched with varying concentrations of 2,4-dichlorophenoxyacetic acid (2,4-D) or α-naphthaleneacetic acid (NAA) alone or in combination with BAP (6-benzylaminopurine). The callus initiation was observed within 2 weeks of inoculation, and subculturing was performed every 3 weeks to maintain the culture. The callus induction rate (%) was recorded after 4 weeks of culture. After 4–5 weeks, the proliferative calli were transferred to MS added with different concentrations of BAP (1.0–3.0 mg L^−1^) and NAA (0.1–1.0 mg L^−1^) for shoot regeneration (indirect organogenesis). The shoot regeneration percentage (%) and the mean number of shoots/callus were noted after 4 weeks. Twenty-four test tubes were taken for each treatment, and every experiment was repeated thrice.

### 4.3. Root Induction and Acclimatization

The callus-derived healthy shoots were inoculated on root induction medium containing different auxins for root development. Varied concentration ranges of indole-3-butyric acid (IBA) and NAA were applied alone or in combinations. The root induction frequency (%) and the mean root number/shoot were noted after 4 weeks. The rooted plantlets were shifted to pots containing a 1:1 ratio of soil and soilrite for acclimatization and were allowed to grow under a greenhouse environment with a temperature and relative humidity of 27 ± 2 °C and 55–60%, respectively.

### 4.4. Extract Preparation and GC–MS Analysis

Fresh and young leaves of in vivo- and in vitro-derived plants were collected. The leaves were air dried and crushed into fine powder in liquid nitrogen using a mortar and pestle, and subsequently, soxhlet extraction was performed using methanol until the powder was completely utilized. This procedure was repeated twice after shaking the volume with the solvent in an orbital shaker for 24 h. The purification of extracts was performed using a syringe filter (0.22 μm, Genetix, New Delhi, India), and 1–2 μL of the extracts was taken as the injecting volume. Gas chromatography–mass spectrometry (GC–MS) analysis was carried out in a gas chromatograph system (model 7890A, Agilent 19091-433HP, Santa Clara, CA, USA) coupled with a mass spectrophotometer fitted with a HP-5 MS fused silica column (5% phenyl methyl siloxane 30.0 m × 250 μm, film thickness 0.25 μm) and interfaced with a 5675C Inert MSD with Triple-Axis detector. Helium gas was used as the carrier gas and was adjusted to a column velocity flow of 1.0 mL/min. The other GC–MS conditions were an ion-source temperature of 250 °C; an interface temperature of 300 °C; a pressure of 16.2 psi; an out time of 1.8 mm; and a 1 μL injector in split mode with a split ratio of 1:50 with an injection temperature of 300 °C. The relative percent amount of each component was calculated by comparing its average peak area to total areas.

### 4.5. Elicitation Treatments

Cadmium chloride (CdCl_2_) and chitosan were purchased from Sigma Aldrich, St. Louis, MO, USA and were used as the abiotic and biotic elicitors. The in vitro-derived plant parts, such as the callus, leaves and roots of *T. indica*, were exposed to five different concentrations of CdCl_2_ (0, 0.1, 0.2, 0.3 and 0.4 mg L^−1^, abbreviated as T0, T1, T2, T3 and T4) and chitosan (0, 1, 5, 25 and 50 mg L^−1^, abbreviated as T0, T1, T2, T3 and T4) augmented MS medium. All the treated samples were processed for further analysis post two-week period. Each elicitation treatment was administered in two conical flasks (50 mL), and every experiment was repeated thrice.

### 4.6. Biochemical Analysis

#### 4.6.1. Sugar Content

The total sugar content was measured following the protocol given by Dey [53]. Fresh tissues (0.5 g), like the callus, leaves and roots, were extracted twice with 90% ethanol. Double-distilled water was added to the extract to obtain a final volume of up to 25 mL. A total of 5.0 mL of concentrated sulfuric acid and 1.0 mL of 5% phenol were added to 1.0 mL of aliquot. Finally, at 490 nm, the sample’s optical density was measured. As a standard, a solution of 1.5 mL of 55% glycerol, 0.5 mL ninhydrin and 4.0 mL double-distilled water was used.

#### 4.6.2. Protein Content

The Bradford [54] method was applied to determine the total protein content of all the samples, wherein 500 mg of samples (fresh weight) of each tissue were homogenized in a prechilled mortar and pestle containing 1.5 mL (0.1 M) phosphate buffer (pH 7.0), and the mixture was centrifuged at 5000× *g* rpm for 10 min. The samples were again centrifuged at 5000× *g* rpm for 10 min after adding 0.5 mL of trichloroacetic acid (TCA). The suspended pellet was rinsed with chilled acetone, and the supernatant was discarded, which was later dissolved in 1.0 mL of 0.1 N sodium hydroxide (NaOH). A total of 0.5 mL of Bradford’s reagent was added to the 0.1 mL of the aliquot before the optical density was measured at 595 nm. Bovine serum albumin (BSA) was taken as the standard.

#### 4.6.3. Proline Content

The Bates et al. [55] method was undertaken to assess free proline. Approximately 200 mg of callus, leaves and root were ground in 5.0 mL of 3.0% aqueous sulfosalicylic acid. The debris was eliminated by centrifuging the mixture at 5000× *g* rpm for 10 min. After the addition of 1.0 mL glacial acetic acid and 1.0 mL ninhydrin in 1.0 mL of extract, the mixture was boiled at 100 °C for 1 h. The mixture was placed in an ice bath, and each sample was extracted with 2.0 mL of toluene. L-proline was used as a standard to determine the amount of light absorbed at the 520 nm wavelength by the finally collected upper organic layer.

#### 4.6.4. Antioxidant Enzyme Assay

Tissues (callus, leaves and roots) of approximately 0.1 g each of *T. indica* were homogenized in a mortar and pestle containing a pre-chilled extraction buffer to evaluate the activity of antioxidant enzymes (0.5 M Na-phosphate, pH 7.0, 3 mM EDTA, 1%PVP, 1%Triton X-100). The homogenate was centrifuged for 20 min at 10,000 rpm (4 °C). The catalase (CAT; EC 1.11.1.6), superoxide dismutase (SOD; EC 1.15.1.1), and ascorbate peroxidase (APX; EC 1.11.1.11) activities were analyzed. The CAT activity was measured according to the method described by Aebi [56]. It was measured as the reduction in absorbance at 240 nm by estimating the rate of H_2_O_2_ decomposition. One unit of CAT is the quantity of CAT required to decompose 1.0 mol of H_2_O_2_/min. The CAT activity was measured using the molar extinction coefficient of 0.036 m M^−1^ cm ^−1^ and expressed as EU mg ^−1^ protein/min. The SOD activity was determined using a technique reported by Dhindsa et al. [57]. The capacity of the supernatant to block the photochemical reaction of nitrobluetetrazolium (NBT) chloride was utilized as the basis for measuring the SOD in the supernatant (NBT). The extract absorbance was measured at 560 nm, and one unit of enzyme activity is equal to the percentage of color that disappeared after being exposed to light for one minute. This value was reported as EU mg^−1^ protein min^−1^.The method developed by Nakano and Asada [58] was undertaken to determine the level of APX activity. The decrease in absorbance at 290 nm, occurring as a result of ascorbic acid oxidation, was used to determine its activity level. An absorption coefficient of 2.8 mM^−1^ cm^−1^ was taken to reach at an estimate of the enzyme’s activity. Its activity was represented in EU mg ^−1^ protein min^−1^, which is calculated as the lowest quantity of enzyme required to digest 1.0 mol of ascorbate per minute. One unit and the activity are measured in EUmg^−1^ protein min^−1^.

#### 4.6.5. Quantification of α-Amyrin Using High-Performance Thin Layer Chromatography (HPTLC)

One (1.0) mg of α-amyrin was dissolved in 1.0 mL of methanol to obtain a standard stock solution of 1 mg/mL. From the stock solution, a series of volumes (0.3, 0.6, 0.9, 1.2, 1.5 and 1.8 μL) were applied on 20 × 10 cm TLC silica plates for standard plot formation. The callus, leaves and roots of the control and elicitor (CdCl_2_ and chitosan)-treated plants of *T. indica* were shade dried for 4 weeks. The dried material was ground into fine powder using a mortar and pestle. The extraction was performed with maceration of 100 mg of powdered sample in 1.0 mL of solvent (methanol:water) in the ratio of 9:1 with continual stirring. The homogenate was centrifuged at 13,000 rpm for 25 min to remove any impurities. The extract was concentrated to a residue with the help of a rotary evaporator at 40 °C; the residue was finally dissolved in solvent and syringe-filtered through a 0.45 μm membrane before further use. HPTLC was performed on 20 × 10 cm aluminum plates coated with a 0.2 μm silica gel layer (60 F254, Merck, Rahway, NJ, USA); these plates were washed with methanol before use to remove impurities and oven dried at 100 °C for 5–10 min. Different volumes of samples and standard were applied with a constant rate of 80 mL/s and a constant flow of nitrogen gas with a band width of 5 mm on sample applicator Linomat V (CAMAG, Muttenz, Switzerland) equipped with a 100 μL syringe. After sample application, the plates were dried at room temperature and developed in a CAMAG twin-trough glass chamber (20 × 10 cm) saturated with mobile phase for 1 h with linear ascending mode up to 90 mm. The mobile phase used in this study was a mixture of solvents (toluene:ethyl acetate) in the ratio of 9.5:0.5. The developed plates were scanned at a wavelength of 510 nm with a TLC scanner V (CAMAG, Muttenz, Switzerland) at a slit dimension of 6.0 × 0.1 mm and scanning speed of 20 mm/s. The peak areas of three replicate samples were used for the quantification of compounds while using the standard peak as the reference.

### 4.7. Statistical Analysis

The obtained data of the various experiments were placed under statistical analysis to assess and verify the reproducibility of the experimental results. All the investigations of the current study were set up in a completely randomized design (CRD). The influence of the abiotic and biotic elicitors, the callus induction and growth, the biochemical attributes, the antioxidant enzyme activities, and the yield and enhancement of α-amyrin were analyzed statistically. The bars in the figures represent the standard error (SE) of the mean, and the data in the tables and figures are the means of three replicates of experiments, which were performed at least twice. The data were subject to analysis of variance (ANOVA) using SPSS software SPSS v. 16 (SPSS Inc., Chicago, IL, USA). The mean values were separated using Duncan’s multiple range test (DMRT) at *p* ≤ 0.05, considered as statistically significant.

## 5. Conclusions

The present study, for the first time, described the GC–MS-based metabolite profiles of in vivo- and in vitro-grown plants of *T. indica*, which revealed the presence of several pharmaceutically important bioactives, like α-amyrin, lupeol, septicine, tocopherol, sitosterol, phytol, etc. The current investigation also discussed successful elicitation and enhancement of the medicinally important triterpenoid α-amyrin by using cadmium chloride and chitosan. With elevated levels of both elicitors (abiotic and biotic), the yield of α-amyrin increased. In response to elicitation, stress-responsive attributes, like sugar, proline and protein, were accumulated more in the tissues, and the antioxidant scavenging ability was upregulated. The content of α-amyrin was quantified through HPTLC, which showed an enhanced yield in the different tissues. Thus, the optimized elicitation technique may be employed as a beneficial tool for the large-scale production and enrichment of important phytoconstituents for therapeutic, commercial applications.

## Figures and Tables

**Table 1:** Callus induction, indirect organogenesis percentage and mean no. of shoots/culture in different concentrations and combinations of 2,4-D-, BAP- and NAA-amended MS medium from leaf explant of *T. indica*.

PGRs (mg L^−1^)			Callus Induction (%)	Organogenesis (%)	
2,4-D	BAP	NAA	Leaf Explant	Shoot Formation (%)	Mean No. of Shoot/ Callus Mass
0.5			68.31 ± 0.88 c	0	0
1.0			76.43 ± 0.91 b	0	0
1.5			82.25 ± 0.41 a	0	0
2.0			68.81 ± 0.98 c	0	0
	1	0.1	56.22 ± 1.24 d	44.13 ± 3.88 d	2.60 ± 0.12 c
	1	0.5	51.32 ± 1.15 e	51.40 ± 3.20 d	2.79 ± 0.14 c
	1	1.0	47.19 ± 0.99 f	39.33 ± 3.21 e	2.32 ± 0.07 c
	2	0.1	27.68 ± 0.61 g	63.50 ± 2.90 b	3.34 ± 0.03 b
	2	0.5	20.11 ± 0.91 h	68.15 ± 4.55 a	4.19 ± 0.15 a
	2	1.0	0	56.10 ± 4.51 c	3.21 ± 0.10 b
	3	0.1	0	37.50 ± 3.79 e	1.90 ± 0.08 d
	3	0.5	0	34.70 ± 4.40 e	1.72 ± 0.09 d
	3	1.0	0	30.36 ± 2.70 f	1.48 ± 0.01 e

The data were scored after 4 weeks of culture. Values are means ± standard error (n = 24). Mean values within a column followed by different letters are significantly different at *p* ≤ 0.05 according to DMRT.

**Table 2:** Effect of different IBA and NAA combinations and concentrations on root induction from in vitro-raised micro shoots of *T. indica* in MS medium.

PGR (mg L^−1^)		% Root Induction	Mean No. Roots/ Shoots
IBA	NAA		
0.5		68.31 ± 1.18 c	7.01 ± 0.01 c
1.0		83.14 ± 1.57 a	8.12 ± 0.02 a
1.5		78.03 ± 0.56 b	7.92 ± 0.01 b
	0.25	31.52 ± 1.24 h	2.47 ± 0.01 i
	0.5	47.57 ± 1.29 f	4.77 ± 0.03 g
	1.0	34.37 ± 1.11 g	3.16 ± 0.02 h
1.0	0.25	58.41 ± 1.18 e	6.02 ± 0.01 e
1.0	0.5	63.35 ± 1.06 d	6.89 ± 0.02 d
0.5	1.0	55.64 ± 1.23 e	5.32 ± 0.01 f

The data were scored after 4 weeks of inoculation. Values are means ± standard error (n = 24). Means followed by different letters in each column are significantly different at *p* ≤ 0.05 using Duncan’s multiple range test (DMRT).

**Table 3:** List of phytocompounds obtained with GC–MS of methanolic leaf extract of the in vivo plant of *T. indica*.

Peak	R. Time	Area	Area%	Name of Compound
1	4.444	58413741	7.20	4h-pyran-4-one, 2,3-dihydro-3,5-dihydroxy-6-methane
2	6.582	2895162	0.36	2-methoxy-4-vinylphenol
3	8.893	12285838	1.52	ethanone, 1-(4-hydroxy-3-methoxyphenyl)
4	9.607	2496143	0.31	1,6,10-dodecatrien-3-ol
5	9.753	2661528	0.33	benzamide
6	10.952	1948509	0.24	3-cyclohexen-1-ol
7	11.429	2135516	0.26	2,6,10-dodecatrien-1-ol
8	11.598	6546426	0.81	6-(p-tolyl)-2-methyl-2-heptenol
9	11.922	2922553	0.36	(r,z)-2-methyl-6-(4-methylcyclohexa-1,4-dien-1-yl)
10	11.999	1632825	0.20	spiro[androst-5-ene-17,1′-cyclobutan]-2′-one
11	12.277	1760491	0.22	phenol, 2,4,5-trimethoxy-3-methane
12	12.333	4624633	0.57	2(4h)-benzofuranone
13	12.647	6157467	0.76	neophytadiene
14	13.866	96537127	11.91	inositol
15	14.147	128600912	15.86	n-hexadecanoic acid
16	15.394	68133736	8.40	phytol
17	15.867	224738398	27.72	9,12,15-octadecatrienoic acid
18	16.010	74847773	9.23	[dodecanoyl(methyl)amino]acetic acid
19	16.939	965421	0.12	carbonic acid, 2-dimethylaminoethyl isobutyl ester
20	17.286	1059839	0.13	9-octadecenal, (z)
21	17.684	879834	0.11	9-octadecenoic acid (z)
22	18.413	1611301	0.20	3-cyclopentylpropionic acid, 2-dimethylaminoethyl ester
23	18.899	4168486	0.51	hexadecanoic acid, 2-hydroxy-1-(hydroxymethyl)ethyl ester
24	20.063	3893883	0.48	1,13-tetradecadiene
25	20.336	4435408	0.55	methyl (z)-5,11,14,17-eicosatetraenoate
26	20.900	1621582	0.20	9-octadecenamide, (z)
27	21.021	2253178	0.28	squalene
28	21.270	648175	0.08	α tocospiro a
29	21.422	842781	0.10	α tocospiro b
30	21.595	5338594	0.66	indolizine, 7-(3,4-dimethoxyphenyl)-1,2,3,5,8,8
31	22.374	727963	0.09	ergosta-5,22-dien-3-ol, (3.beta.,22e,24s)
32	22.529	5792185	0.71	(+)-septicine
33	22.785	607484	0.07	Gamma tocopherol
34	23.025	1455700	0.18	stigmasta-5,22-dien-3-ol, acetate, (3.beta.)-
35	23.409	4931963	0.61	vitamin E
36	24.482	11552025	1.42	ergost-5-en-3-ol
37	24.730	5072544	0.63	stigmasta-5,22-dien-3-ol
38	25.424	26109537	3.22	Gamma sitosterol
39	25.972	3018664	0.37	4,4,6a,6b,8a,11,11,14b-octamethyl-1,4,4a,5,6,6a,6
40	26.430	2263070	0.28	lanosterol
41	26.604	16351452	2.02	α amyrin
42	27.823	1929876	0.24	lupeol
43	28.292	2844723	0.35	phytyl decanoate
44	31.950	3036051	0.37	isopropyl linoleate

**Table 4:** List of phytocompounds obtained with GC–MS analysis of methanolic leaf extract of in vitro-regenerated plants of *T. indica*.

Peak	R. Time	Area	Area%	Name of Compound
1	4.419	44896275	9.14	1,5-anhydro-6-deoxyhexo-2,3-diulose
2	8.915	16295910	3.32	ethanone, 1-(4-hydroxy-3-methoxyphenyl)
3	9.983	2665097	0.54	1,2-benzenedicarboxylic acid
4	10.860	449741	0.09	1-(4-isopropylphenyl)-2-methylpropyl acetate
5	11.330	2313565	0.47	2-(2-iodo-ethyl)-1,3,3-trimethyl-cyclohexene
6	11.427	1709951	0.35	2,6,10-dodecatrien-1-ol, 3,7,11-trimethane
7	11.596	5409117	1.10	6-(p-tolyl)-2-methyl-2-heptenol
8	11.920	2398279	0.49	(r,z)-2-methyl-6-(4-methylcyclohexa-1,4-dien-1-yl)
9	11.996	1228296	0.25	spiro[androst-5-ene-17,1′-cyclobutan]-2′-one
10	12.327	4067868	0.83	2(4h)-benzofuranone
11	12.449	1022344	0.21	2-cyclohexen-1-one
12	12.644	8962082	1.82	neophytadiene
13	12.970	369300	0.08	1,2-benzenedicarboxylic acid,
14	13.095	4317055	0.88	3,7,11,15-tetramethyl-2-hexadecen-1-ol
15	13.388	22914178	4.66	inositol
16	13.571	346778	0.07	eicosanoic acid, methyl ester
17	14.115	92093974	18.75	n-hexadecanoic acid
18	15.271	722974	0.15	11,14,17-eicosatrienoic acid, methyl ester
19	15.381	39588281	8.06	phytol
20	15.829	141327110	28.77	9,12,15-octadecatrienoic acid, (z,z,z)
21	15.970	9835497	2.00	9-octadecenoic acid (z)
22	17.406	16012414	3.26	n-benzyl-p-toluene sulfonamide
23	18.408	703718	0.14	3-cyclopentylpropionic acid, 2-dimethylaminoethyl ester
24	18.659	3753517	0.76	2-methyltetracosane
25	18.895	1471172	0.30	hexadecanoic acid, 2-hydroxy-1-(hydroxymethyl)
26	18.968	820931	0.17	di-n-octyl phthalate
27	20.030	921001	0.19	ethyl (9z,12z)-9,12-octadecadienoate
28	20.097	664029	0.14	9,12,15-octadecatrienoic acid
29	20.894	1868283	0.38	9-octadecenamide
30	21.270	538175	0.19	α tocospiro a
31	21.422	782781	0.21	α tocospiro b
32	21.584	2285700	0.47	indolizine
33	21.654	1131106	0.23	octacosanol
34	21.837	412305	0.08	hexacosanoic acid, methyl ester
35	22.516	2113879	0.43	(+)-septicine
36	22.778	563696	0.11	Gamma tocopherol
37	23.021	1013780	0.21	stigmasta-5,22-dien-3-ol
38	23.396	2252853	0.46	vitamin E
39	24.468	6169960	1.26	ergost-5-en-3-ol
40	24.714	3149052	0.64	stigmasta-5,22-dien-3-ol
41	25.400	15934409	3.24	Gamma sitosterol
42	25.730	1261223	0.26	dl-2-phenyltryptophane
43	25.953	1345166	0.27	4,4,6a,6b,8a,11,11,14b-octamethyl-1,4,4a,5,6,6a,6b,7,8,8a,
44	26.583	9789411	4.13	α amyrin
45	27.618	1396959	0.58	lupeol
46	28.272	1633211	0.33	phytyltetradecanoate
47	30.285	2595029	0.53	3.beta.-myristoylolean-12-en-16.beta.-ol
48	31.909	1795754	0.37	isopropyl linoleate
49	32.203	1231679	0.25	9,12-octadecadienal, dimethyl acetate
50	32.658	2943911	0.60	chromium

**Table 5:** Accumulation of α-amyrin content (µg g^−1^ DW) in leaf, callus and root parts of *Tylophora indica* on different treatments of CdCl_2_.

Plant Parts Used	T0	T1	T2	T3	T4
Leaf	1.78 ± 0.03 e	1.99 ± 0.02 d	2.21 ± 0.03 c	2.72 ± 0.01 a	2.56 ± 0.02 b
Callus	0.98 ± 0.02 e	1.11 ± 0.03 d	1.26 ± 0.03 c	1.51 ± 0.02 a	1.38 ± 0.02 b
Root	0.25 ± 0.02 e	0.31 ± 0.03 d	0.45 ± 0.02 c	0.68 ± 0.02 a	0.53 ± 0.03 b

T0 = Control, T1 = 0.1 mg L^−1^, T2 = 0.2 mg L^−1^, T3 = 0.3 mg L^−1^, T4 = 0.4 mg L^−1^. Values are mean ± standard deviation of three experiments with two replicates each (n = 2). Mean values in each row followed by different letters are significantly different at *p* = 0.05 according to DMRT.

**Table 6:** Accumulation of α-amyrin content (µg g^−1^ DW) in leaf, callus and root parts of *Tylophora indica* on different treatments of chitosan.

Plant Parts Used	T0	T1	T2	T3	T4
Leaf	1.67 ± 0.02 e	1.88 ± 0.03 d	2.11 ± 0.03 c	2.64 ± 0.02 a	2.37 ± 0.01 b
Callus	0.76 ± 0.03 e	1.08 ± 0.02 d	1.19 ± 0.01 c	1.45 ± 0.02 a	1.31 ± 0.01 b
Root	0.18 ± 0.02 e	0.29 ± 0.02 d	0.40 ± 0.02 c	0.61 ± 0.03 a	0.51 ± 0.02 b

T0 = Control, T1 = 1 mg L^−1^, T2 = 5 mg L^−1^, T3 = 25 mg L^−1^, T4 = 50 mg L^−1^. Values are mean ± standard deviation of three experiments with two replicates each (n = 2). Mean values in each row followed by different letters are significantly different (at *p* = 0.05) according to DMRT.

## Data Availability

Data are contained within the article.
